# Crop‐to‐wild hybridization in cherries—Empirical evidence from *Prunus fruticosa*

**DOI:** 10.1111/eva.12677

**Published:** 2018-07-26

**Authors:** Lenka Macková, Petr Vít, Tomáš Urfus

**Affiliations:** ^1^ Department of Botany Faculty of Science Charles University Prague Czech Republic; ^2^ Institute of Botany The Czech Academy of Sciences Průhonice Czech Republic; ^3^ Faculty of Environmental Sciences Czech University of Life Sciences Prague Prague Czech Republic

**Keywords:** absolute genome size, cherry, crop‐to‐wild gene flow, hybridization, introgression, ploidy level, *Prunus*

## Abstract

Crop cultivation can lead to genetic swamping of indigenous species and thus pose a serious threat for biodiversity. The rare Eurasian tetraploid shrub *Prunus fruticosa* (ground cherry) is suspected of hybridizing with cultivated allochthonous tetraploid *P. cerasus* and autochthonous diploid *P. avium*. Three *Prunus* taxa (447 individuals of *P. fruticosa*, 43 of *P. cerasus* and 73 of *P. avium*) and their hybrids (198 individuals) were evaluated using analysis of absolute genome size/ploidy level and multivariate morphometrics. Flow cytometry revealed considerable differentiation in absolute genome size at the tetraploid level (average 2C of *P. fruticosa* = 1.30 pg, average 2C of *P. cerasus* = 1.42 pg, i.e., a 9.2% difference). The combination of methods used allowed us to ascertain the frequency of hybrids occurring under natural conditions in Central Europe. The morphological evaluation of leaves was based upon distance‐based morphometrics supplemented by elliptic Fourier analysis. The results provided substantial evidence for ongoing hybridization (hybrids occurred in 39.5% of *P. fruticosa* populations). We detected homoploid introgressive hybridization with alien *P. cerasus* at the tetraploid level. We also found previously overlooked but frequent triploid hybrids resulting from heteroploid hybridization with indigenous *P. avium*, which, however, probably represent only the F1 generation. Although both hybrids differ in ploidy, they cannot be distinguished using morphometrics. Hybrids are frequent and may endanger wild populations of genuine *P. fruticosa* via direct niche competition or, alternatively or in addition, via introgression at the homoploid level (i.e., genetic swamping). The cultivation of cherries thus substantially threatens the existence of genuine *P. fruticosa*.

## 1 INTRODUCTION

Human activities significantly contribute to the reduction in global plant diversity (e.g., Frankham, Ballou, & Briscoe, 2010). Intensively studied phenomena such as degradation accompanied by fragmentation of natural habitats usually cause changes in the distribution of species, including extinction events or invasions (Corlett, 2016). However, the adverse effects of hybridization on plant diversity have scarcely been evaluated (Ellstrand & Elam, 1993; Levin, Francisco‐Ortega, & Jansen, 1996; Rhymer & Simberloff, 1996; Todesco et al., 2016). Besides invasive taxa (e.g., Hejda, Pyšek, & Jarošík, 2009), hybridization with commercial crops poses a significant threat to indigenous species (Ellstrand, Prentice, & Hancock, 1999). The potential repercussions of hybridization have been repeatedly demonstrated (Todesco et al., 2016). However, even though commercial crops are ubiquitous, the topic of crop‐to‐wild hybridization has been addressed by relatively few empirical studies (e.g., Aerts et al., 2013; Arrigo et al., 2011).

Hybridization as an evolutionary process (together with polyploidization) significantly contributes to the diversity of vascular plants (Soltis & Soltis, 2009). It may lead to evolutionary novelties and the establishment of new species. On the other hand, when reproduction barriers leak, hybridization followed by backcrossing may lead to the extinction of parental species (Rhymer & Simberloff, 1996). The production of hybrid seeds increases, and the reproduction success of parental species is significantly reduced (Levin et al., 1996). Hybrids with the same or greater fitness as their parental species can significantly affect the populations of their parents (genetic swamping; Todesco et al., 2016). Last but not least, even the mere production of sterile hybrid individuals may lead to the extinction of rare parents through the wasteful production of maladapted hybrids, which decreases the number of potential mating partners, and by competition for resources and suitable niches (i.e., demographic swamping; Todesco et al., 2016).

Some rare (i.e., low abundance) species can hybridize with their widespread congeners (e.g., introgression of *Morus* L., Burgess, Morgan, Deverno, & Husband, 2005; *Rumex* L., Ruhsam, Jacobs, Watson, & Hollingsworth, 2015), which in extreme cases may lead to local extinction as a result of demographic or genetic swamping (Ellstrand & Elam, 1993; Todesco et al., 2016). Introgressive hybrid swarms typically occur in transitional or peripheral habitats (e.g., Čertner, Kolář, Schönswetter, & Frajman, 2015; Raudnitschka, Hensen, & Oberprieler, 2007). In addition, anthropogenic activities may promote the formation of hybrid swarms by enhancing secondary contact between species (e.g., Hanušová, Ekrt, Vít, Kolář, & Urfus, 2014) or by creating open habitats suitable for the survival and expansion of hybrids (Wójcicki, 1991). Hybridization with ubiquitously cultivated commercial, ornamental and consumer plants poses a threat to some indigenous species (Ellstrand et al., 2013).

Crop‐to‐wild gene flow has been documented in several indigenous plant species and may lead to the establishment of aggressive weeds or even the extinction of rare species (Ellstrand et al., 2013, 1999). So far, only a few human‐induced (i.e., with the participation of crop plants) cases of hybridization have been reported. Spontaneous introgression of wild *Prunus orientalis* (Duhamel) by cultivated *Prunus dulcis* (Mill.) D. A. Webb in south‐west Asia (Delplancke et al., 2012) and genetic erosion of the rare wild species *Malus sylvestris* (L.) Mill. in Belgium by domesticated apple (*Malus domestica* Borkh.; Coart, van Glabeke, de Loose, Larsen, & Roldán‐Ruiz, 2006) often serve as model examples. One extreme case of crop‐to‐wild gene flow is the genus *Aegilops* L. in the Mediterranean, where more than one quarter of some wild populations bear signs of introgression from wheat (Arrigo et al., 2011). Besides conservation consequences, genetic swamping of wild relatives via hybridization with crops can lead to tremendous economic losses because wild taxa serve as an essential gene pool resource for breeding programmes (Barać et al., 2017; Ganopoulos, Aravanopoulos, & Tsaftaris, 2013).

A prime example of a species endangered by human‐induced gene flow from cultivated crops is *Prunus fruticosa* Pall. (ground cherry), a rare and morphologically variable relict Eurasian shrub of steppes and forest steppes (Jäger & Seidel, 1995; Meusel, Jäger, & Weinert, 1965; Rhodes & Maxted, 2016). It is tetraploid (2*n* = 32 chromosomes; Oldén & Nybom, 1968; Scholz & Scholz, 1995) and self‐incompatible (also propagated by root shoots; Pruski, 2007; Scholz & Scholz, 1995). *Prunus fruticosa* is of potentially considerable importance in cherry breeding programmes, as it possesses suitable characters for growing in steppe conditions (Barać et al., 2017; Dzhangaliev, Salova, & Turekhanova, 2003; Iezzoni, 2008; Iezzoni & Mulinix, 1992; Pruski, 2007). Widely cultivated sour and sweet cherries (*Prunus cerasus* L. and *Prunus avium* (L.) L.) are close relatives of *P. fruticosa* and easily hybridize with it (e.g., Scholz & Scholz, 1995). Whereas diploid *P. avium* is an indigenous European taxon (2*n* = 16; Jäger & Seidel, 1995; Marhold & Wójcicki, 1992; Webb, 1968), tetraploid *P. cerasus* in Europe is an alien species that occasionally escapes from cultivation (e.g., Scholz & Scholz, 1995; Webb, 1968). *Prunus cerasus* has been proven to be an allotetraploid that has originated through hybridization of *P. fruticosa* and *P. avium* (2*n* = 32; Horvath, Zanetto, Christmann, Laigret, & Tavaud, 2008; Oldén & Nybom, 1968; Schuster & Schreibner, 2000; Tavaud, Zanetto, David, Laigret, & Dirlewanger, 2004).

The enormous morphological variation of *Prunus fruticosa* has been repeatedly ascribed to interspecific hybridization (e.g., Chrtek, 1992; Scholz & Scholz, 1995). On the basis of morphology (the purported discriminative characters being plant height and hairs on the abaxial surface of the lamina), two types of hybrids have been described (Lepší, Lepší, Boublík, & Kolář, 2011; Wójcicki, 1988). One of them, *Prunus* × *eminens* Beck (*P. fruticosa* × *P. cerasus*; 2*n* = 4× = 32; Scholz & Scholz, 1995; Webb, 1968; Wójcicki, 1991), has been reported to be abundant (35% of hybrids estimated in the Czech Republic and Slovakia; Wójcicki & Marhold, 1993) and partly fertile (Macková, Vít, Ďurišová, Eliáš, & Urfus, 2017) whereas the other, *Prunus* ×*mohacsyana* Kárpáti (*P. fruticosa* × *P. avium*; 2*n* = 3× = 24; (Marhold & Wójcicki, 1992; Oldén & Nybom, 1968), has been recorded only extremely rarely (Macková et al., 2017; Scholz & Scholz, 1995; Wójcicki & Marhold, 1993) and has been confirmed to be sterile (Macková et al., 2017). Thus, hybridization appears to be a major threat to *P. fruticosa* that is directly connected with human activities such as the cultivation of cherries (Boratyński, Lewandowska, & Ratyńska, 2003; Wójcicki, 1991; Wójcicki & Marhold, 1993).

In contrast to morphology, which has hitherto been used to indicate *P. fruticosa* hybridization, nuclear DNA content represents a highly reproducible species‐specific marker (Loureiro et al., 2010) and is convenient for the delimitation of *Prunus* taxa because particular species differ in their ploidy level or absolute genome size (e.g., Baird, Estager, & Wells, 1994; García‐Verdugo et al., 2013; Macková et al., 2017; Maghuly, Schmoellerl, Temsch, & Laimer, 2010). Without the use of additional markers (e.g., genome size or ploidy level), it is often difficult to accurately identify hybrids and pure individuals based on morphology only (Ruhsam et al., 2015; Vítová, Vít, & Suda, 2015), and this can result in the misled protection of hybrid populations (Kabátová, Vít, & Suda, 2014; Vít, Wolfová, Urfus, Tájek, & Suda, 2014).

The main goal of this study was to examine the extent of interspecific hybridization of the rare species *Prunus fruticosa* with wild and cultivated cherries (*P. cerasus* and *P*. *avium*) and to evaluate the impact of hybridization on pure *Prunus fruticosa* populations in Central Europe. To meet this goal, we addressed the following questions: (a) Do ploidy level and absolute genome size correlate with patterns of morphology and delimit *Prunus* taxa on a large spatial scale? (b) What is the frequency of hybrids under natural conditions? and (c) May the presence of hybrids indicate that populations of *P. fruticosa* are under threat from hybridization (incl. introgression)? To find answers to these questions, we collected fresh plant material in natural populations, estimated their nuclear DNA content using flow cytometry and employed distance‐based morphometrics together with elliptic Fourier analysis to describe the variation in short‐shoot leaves.

## 2 MATERIALS AND METHODS

### 2.1 Sampling

**Figure 1:**
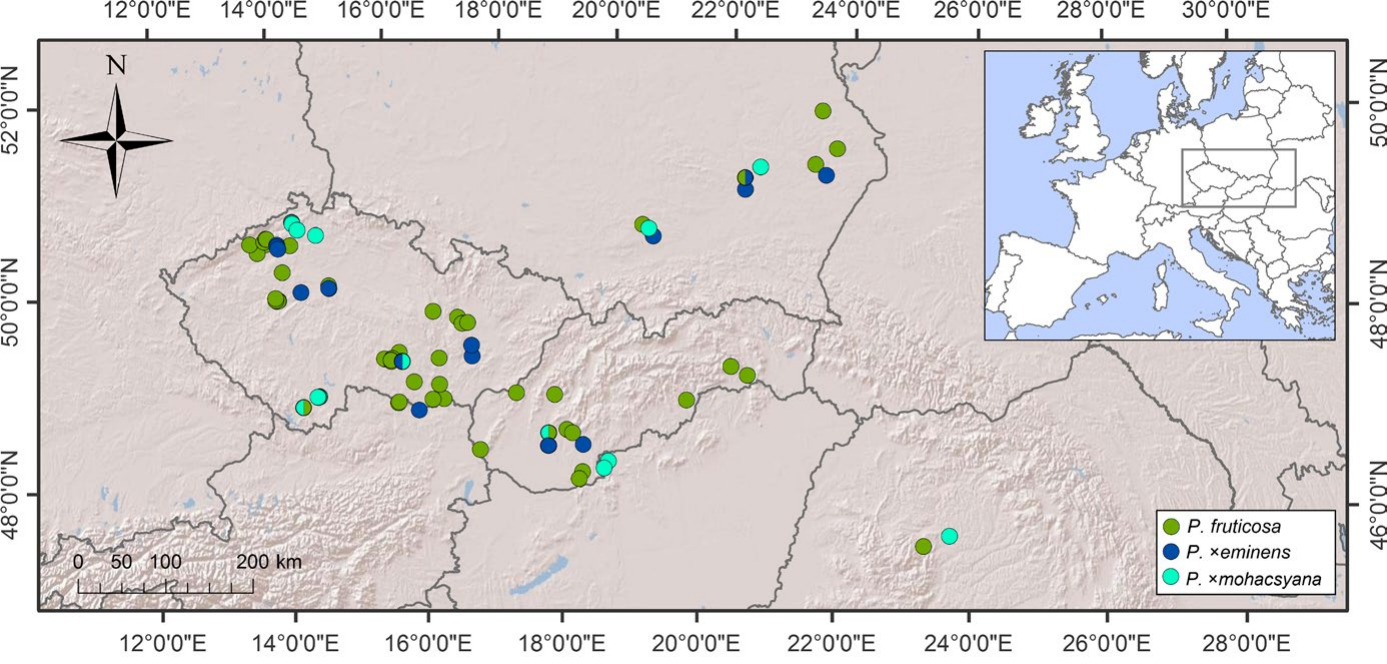
Sample locations of *Prunus fruticosa* and its hybrids in Central Europe

Samples from the Central European area (76 populations—46 *Prunus fruticosa*, 12 *Prunus* × *mohacsyana*, 10 *Prunus* × *eminens*, eight mixed) were collected in 2010–2013 in the Czech Republic (54 populations), Slovakia (13 populations) and Poland (seven populations; marginally also in Romania—two populations; Figure 1, Supporting information Table S1). Samples of the putative parents *Prunus cerasus* (43 individuals from 12 locations) and *Prunus avium* (73 individuals from 38 locations) were also collected in the study area for a better understanding of ongoing microevolutionary processes. Each population sample (usually 5–10 individuals, depending on population size) was represented by a branchlet with vegetative short‐shoot leaves. Sampled individuals were as distant from each other as possible to avoid the collection of clonally emerged individuals. Individuals growing together in one place obviously separated from another place were considered a discrete population. As regards *P. cerasus* and *P. avium*, about three individuals were sampled from each location because these cultivated taxa are scattered in the landscape instead of constituting numerous populations.

The taxa were determined based on their ploidy level (indicating triploid *Prunus* × *mohacsyana* and diploid *P. avium*). Tetraploids were differentiated based on the presence of hairs on the abaxial surface of the lamina (glabrous *P. fruticosa* vs hairy *P*. ×*eminens* and *P. cerasus*) and growth form (shrubby *P. fruticosa* and *P*. ×*eminens* vs tree‐like *P. cerasus*).

In total, plant material from 761 individuals of *Prunus* taxa (447 *P. fruticosa*, 99 *Prunus* × *mohacsyana*, 99 *P*. ×*eminens*, 43 *P. cerasus* and 73 *P. avium*) were used for three types of analyses—absolute genome size analysis using flow cytometry (FCM), distance‐based morphometrics and elliptic Fourier analysis. Dry plant material was used (short‐shoot leaves taped on to sheets of cardboard) for morphometrics, and fresh plant material was necessary for flow cytometric analysis.

### 2.2 Flow cytometry (FCM)

Ploidy levels/absolute genome sizes of 761 individuals (see Supporting information Table S1 for samples details) were estimated using a Partec CyFlow instrument (Partec GmbH, Münster, Germany) equipped with a green solid‐state laser (Cobolt Samba, 532 nm, 100 mW). A slightly modified procedure following Doležel, Greilhuber, and Suda (2007) was adopted for the isolation and of staining nuclei. *Bellis perennis* L. (2C = 3.38 pg; Schönswetter, Suda, Popp, Weiss‐Schneeweiss, & Brochmann, 2007) was used as the internal standard. About 1.5 cm^2^ of fresh laminar tissue together with 1.8 cm^2^ of the internal standard was chopped in 0.5 ml of ice‐cold Otto I buffer (0.1 M citric acid, 0.5% Tween 20; Doležel et al., 2007) in a Petri dish. The suspension was filtered through a 42‐μm nylon mesh filter and incubated for at least 20 min at room temperature. The suspension was then stained by a solution containing 1 ml of Otto II buffer (0.4 M Na_2_HPO_4_·12 H_2_O; Doležel et al., 2007), β‐mercaptoethanol (final concentration of 2 μl/ml), propidium iodide and RNase IIA (both at the final concentrations of 50 μg/ml). Subsequently, stained samples were run through the flow cytometer. Isolated stained nuclei were excited with a laser beam, and the fluorescence intensity of 3,000 particles was recorded.

Because of the significant amounts of secondary metabolites contained in *Prunus* material (typical of the whole Rosaceae), which complicate FCM analyses, certain optimization steps had to be carried out (for details, see Macková et al., 2017). Although most of the samples were measured at one time point only, we checked the stability of FCM measurements over a long time period (from May to August, 18 individuals from three locations). Variation between two different measurements did not exceed 4% (for information on the stability of FCM measurements over short periods, see Macková et al., 2017). The whole range of measured absolute genome size values was calibrated by chromosome counts (standard karyological methodology; e.g., Lepší, Vít, Lepší, Boublík, & Suda, 2008).

Resulting FCM histograms were analysed using FloMax (version 2.4d, Partec, Münster, Germany). Absolute genome size values were visualized as boxplots in PAST 2.17c (Hammer, Harper, & Ryan, 2001) and as scatter plots in Microsoft Excel 2010. One‐way ANOVA followed by Tukey's HSD test in PAST 2.17c (Hammer et al., 2001) was used to ascertain the significance of absolute genome size differences between species.

### 2.3 Distance‐based morphometrics

To examine morphological variation of the *Prunus* taxa under study, 17 characters (13 primary, four ratio)—eight vegetative and nine generative (see Table 1)—were selected based on the literature (Lepší et al., 2011; Wójcicki, 1988, 1991; Wójcicki & Marhold, 1993) and own field observations. Well‐developed short‐shoot leaves (two leaves per individual) and flowers were measured using a digital calliper (accuracy 0.01 mm) and a stereo microscope (Olympus SZ51; magnification 40 × ). Most of the time, only short‐shoot leaves were observed (because of their narrower range of variation; Marhold & Wójcicki, 1992). Plant height was measured in the field. Abaxial hairs were measured on at least four leaves per individual and then averaged. Plant height, shape of laminar tip, adaxial hairs and abaxial hairs were evaluated using semiquantitative scales (see Table 1). In total, 1,422 leaves (see Supporting information Table S1 for samples details) and only 84 flowers were measured because the flowering period was very short. Because *P. fruticosa* scarcely bears fruits (Chudíková, Ďurišová, Baranec, & Eliáš, 2012), no fruits were included in the study.

**Table 1:** List of measured characters on vegetative and generative organs of *Prunus* taxa under study used in distance‐based morphometric analysis

Description of character	Abbreviation	Unit
Plant height	Height	1 = to 50 cm, 2 = 50‐100 cm, 3 = over 100 cm, 4 = tree
Laminar length	Length	mm
Laminar width	Width	mm
Distance from the widest part of the lamina to the laminar tip	Widest to tip	mm
Shape of laminar tip	Tip	1 = obtuse, 2 = obovate, 3 = elliptic with aristate apex, 4 = elliptic with broadly acuminate apex
Adaxial hairs (density of hairs on the adaxial surface of lamina)	Adax hairs	1 = glabrous, 2 = short hairs, 3 = long hairs, 4 = long and also short hairs
Abaxial hairs (density of hairs on the abaxial surface of lamina)	Abax hairs	1 = glabrous, 2 = scattered pubescent, 3 = sparsely pubescent, 4 = densely pubescent
Laminar length/width (ratio of length and width of the lamina)	Length/width	–
Petal length	–	mm
Petal width	–	mm
Hypanthium length	–	mm
Sepal length	–	mm
Sepal width	–	mm
Peduncle length	–	mm
Petal length/width (ratio of length and width of the petal)	–	–
Sepal length/width (ratio of length and width of the sepal)	–	–
Hypanthium length/sepal length (ratio of hypanthium and sepal length)	–	–

The data matrix was evaluated using multivariate statistical methods in PAST 2.17c (Hammer et al., 2001). Basic descriptive statistics, including the minimum, maximum, mean and the 25th and 75th percentile, were computed for each of the vegetative characters of all taxa under study. Principal component analysis (PCA) was employed to visualize the basic structure of the data in Canoco 5 (Ter Braak & Šmilauer, 2012). Absolute genome size was passively projected on to PCA diagrams using a local regression (loess) model in Canoco 5 (Ter Braak & Šmilauer, 2012). Redundancy analysis (RDA; Van den Wollenberg, 1977) with a Monte Carlo permutation test (999 permutations) performed in Canoco 5 (Ter Braak & Šmilauer, 2012) and correlation analysis carried out in R 3.4.3 (R Core Team, 2017; visualized using Microsoft Excel 2010) were used to test for a link between morphological variation (represented by PC1 scores of distance‐based PCA) and absolute genome size.

### 2.4 Elliptic Fourier analysis

Shape contours of 1,407 leaves (see Supporting information Table S1 for samples details) were investigated using elliptic Fourier analysis. Only well‐developed leaves were included in the analysis (15 partly damaged leaves were excluded). Two leaves of each individual were taped on to a sheet of cardboard paper and scanned (scanner Canon MP270 series Printer; 300 dpi). For leaf shape analysis based on elliptic Fourier descriptors (Kuhl & Giardina, 1982), the SHAPE 1.3 package (Iwata & Ukai, 2002) was employed. The leaf shapes were converted into chain codes using ChainCoder, and the CHC2NEF programme converted these chain codes into coefficients of elliptic Fourier descriptors (using 20 harmonic axes). These coefficients were used to calculate the scores of principal components using the PrinComp function. The PrinComp routine also allowed the reconstruction of the leaf shape, corresponding to values of +2 and −2 standard deviations on the first and second component axes (see Lepší, Vít, Lepší, Boublík, & Kolář, 2009; and Macková et al., 2017, for details). The first and second component axes were visualized using Microsoft Excel 2010.

## 3 RESULTS

### 3.1 Absolute genome size and DNA ploidy level

**Figure 2:**
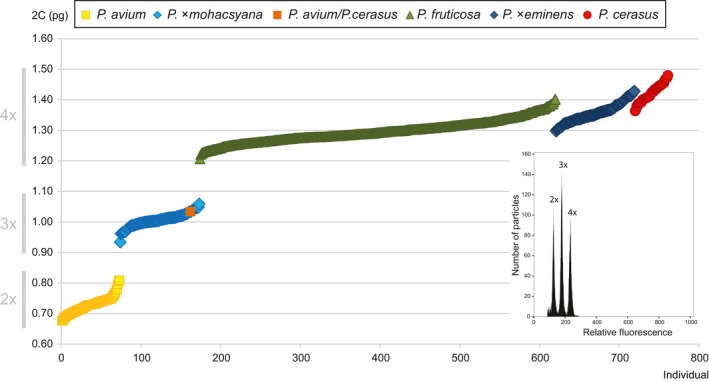
Absolute genome size variation of the five *Prunus* taxa under study. PI‐stained nuclei isolated from 761 leaves. Three ploidy levels were detected: diploid (2×), triploid (3×) and tetraploid (4×). The values are in picograms (pg). Orange‐highlighted individual represent triploid with a well‐developed trunk. A histogram of simultaneous flow cytometric analyses of three ploidy levels is in the right corner. Peak designations: 2× = diploid *P. avium*, 3× = triploid *Prunus* tree form, 4× = tetraploid *P. cerasus*

Ploidy levels and absolute genome sizes of 761 *Prunus* accessions were ascertained by flow cytometry (see Supporting information Table S1 for samples details). Three ploidy levels were detected: diploid (*P. avium*; average 2C = 0.73 pg), triploid (*P*. ×*mohacsyana*; average 2C = 1.01 pg) and tetraploid (*P. fruticosa*,* P*. ×*eminens* and *P. cerasus*; Figure 2, Supporting information Table S2). Moreover, the three tetraploid taxa tended to differ in absolute genome size (*P. fruticosa*—average 2C = 1.30 pg, *P*. ×*eminens*—average 2C = 1.36 pg, *P. cerasus*—average 2C = 1.42 pg, i.e., a 9.2% difference between parental taxa; Supporting information Table S2). Absolute genome size values of tetraploid taxa formed a continuous series of partly overlapping values (Figure 2). Nevertheless, absolute genome size differed significantly between all analysed groups (*F*_4, 755_ = 8826, *p *<* *0.001) as well as between the three tetraploid taxa (*F*_2, 585_ = 311.8, *p *<* *0.001) in ANOVA. Separate Tukey's HSD tests revealed five and three groups for all and for the three tetraploid taxa, respectively.

### 3.2 Distance‐based morphometrics

**Figure 3:**
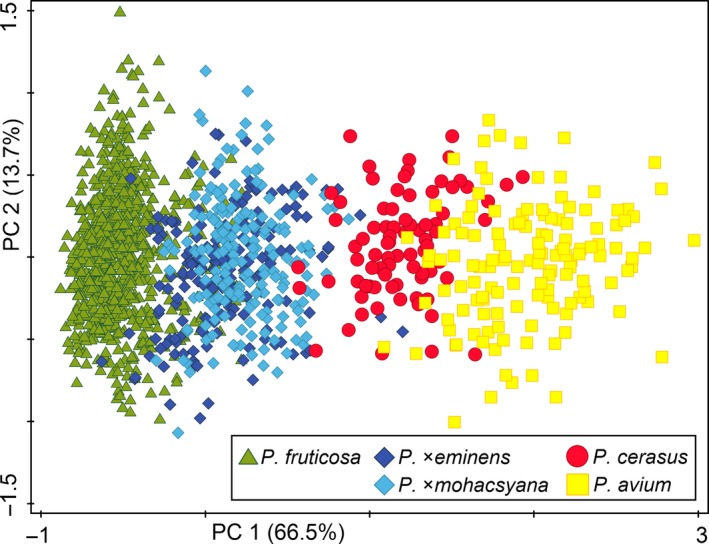
Ordination diagram of principal component analysis using eight vegetative morphological characters of 1,422 leaves of *Prunus* taxa under study

**Figure 4:**
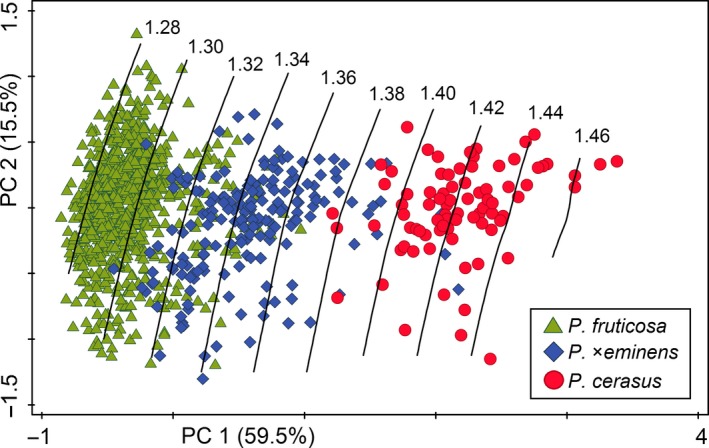
Correspondence of morphological variation and absolute genome size in three *Prunus* taxa studied. Ordination diagram of principal component analysis based on eight vegetative morphological characters of 1,099 leaves of tetraploid *Prunus* taxa. Absolute genome size (values in pg DNA) is passively projected on to the diagram using a local regression (loess) model

**Figure 5:**
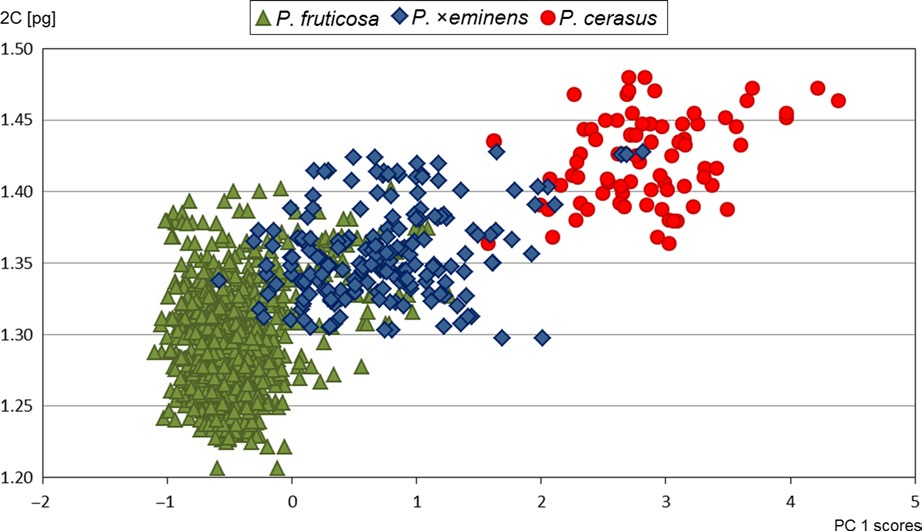
Correlation analysis of tetraploid *Prunus* taxa (1,099 individuals) under study, showing a link between morphology (represented by the first principal component scores) and absolute genome size, explaining 53% of the overall variation (*r *=* *0.729; *t *=* *35.3, *df* = 1097, *p *<* *0.001)

Morphometric variation of 1,422 leaves (see Supporting information Table S1 for samples details) was analysed using distance‐based morphometrics (for descriptive statistics, see Supplementary Table S3). Principal component analysis (PCA) of all five taxa under study, based on eight vegetative characters of leaves, revealed three obvious groups of putative parental taxa: *P. fruticosa, P. cerasus* and *P. avium* (although *P. cerasus* and *P. avium* partly overlapped; Figure 3). The hybrids *P*. ×*mohacsyana* and *P*. ×*eminens* formed a compact, overlapping cluster between their putative parents (the first and the second axes explaining 66.5 and 13.7% of the variation, respectively; Figure 3). The distance from the widest part of the lamina to the laminar tip, laminar width and laminar length was the most tightly correlated (see Supporting information Figure S1) with the first component axis. Thus, the eight vegetative characters measured on leaves could not distinguish between the hybrids. The hybrids grouped together even in the case of PCA using characters on generative organs (84 flowers – 42 *P. fruticosa*, 27 *P*. ×*mohacsyana*, 15 *P*. ×*eminens*; Supporting information Figure S2). PCA of only tetraploid taxa showed clearly distinguished putative parental taxa (*P. fruticosa* and *P. cerasus*) with the hybrid *P*. ×*eminens* scattered between them with a partial overlap (the first and the second axes explaining 59.5 and 15.5% of the variation, respectively; Figure 4). It is important that absolute genome size appeared to be well correlated with the first PCA axis; absolute genome size tended to increase from *P. fruticosa* to *P. cerasus* (see the perpendicularly oriented loess curves in Figure 4). The significant association between leaf morphology and absolute genome size of tetraploid taxa was further confirmed by RDA (*p *=* *0.001, 999 permutations); absolute genome size explained 31.9% of the variation (Supporting information Figure S3A). Five morphological characters (Width, Widest to tip, Length, Abax hairs, Height, Tip) exhibited strong positive correlation with the canonical/genome size axis (see Table 1 for character abbreviations; Supporting information Figure S3B). Moreover, a significant correlation between leaf morphology (represented by PC1 scores) and absolute genome size was found (*r *=* *0.729; *t *=* *35.3, *df* = 1097, *p *<* *0.001), explaining 53% of the overall variation (Figure 5).

### 3.3 Elliptic Fourier analysis

**Figure 6:**
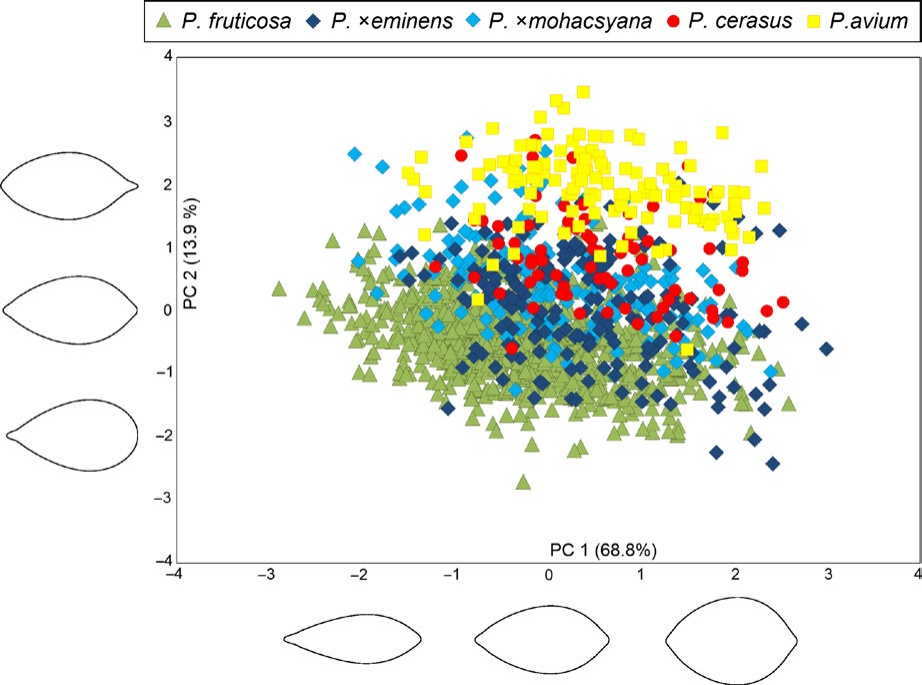
Ordination diagram of principal component analysis of Fourier coefficients describing variability in laminar shape of 1,407 leaves of the *Prunus* taxa under study. PCA scores are standardized to unit variance (units in standard deviation, SD). Reconstructed leaf contours (petiole connection on the left) corresponding to values of −2 SD, 0 and +2 SD are shown along the PC axes

Variation in the shape contours of 1,407 leaves (see Supporting information Table S1 for samples details) was evaluated using elliptic Fourier analysis. The groups of *Prunus* taxa under study overlapped more in comparison with distance‐based morphometrics (Figure 6). *Prunus avium* formed the most differentiated cluster, while the *P. fruticosa* cluster was distinguished only partly. Nevertheless, both overlapped with other *Prunus* taxa in the principal component analysis. On the contrary, *P. cerasus* and both hybrids were scattered between these two clusters and formed a linked and completely overlapping cluster (Figure 6). The first component axis (68.8%) explained the most variation but was not taxonomic specific (variation in relative leaf width), while the second component axis (13.9%), describing variation in the shape of the leaf base and the shape of the leaf tip, reflecting differences between the taxa studied. The most differentiated groups, *P. avium* and *P. fruticosa*, had elliptic leaves with an aristate apex and obovate leaves with an obtuse apex, respectively. *Prunus cerasus*,* P*. ×*eminens* and *P*. ×*mohacsyana* clustered together and tended to form elliptic leaves with a broadly acuminate apex, never obtuse or with an aristate apex (Figure 6). Thus, leaf shape represents a suitable additional character for the determination of parental *Prunus* taxa; however, it fails to distinguish hybrids (similar to distance‐based morphometrics).

### 3.4 Frequency of hybrids under natural conditions

Our multidisciplinary approach has revealed that only 60.5% of populations previously reported to represent genuine *Prunus fruticosa* did not include hybrids; actually, 39.5% of the populations were of hybrid origin (randomly spatially distributed). Most of the hybrid populations under study were composed exclusively of individuals belonging to one of the hybrids; 15.8% of populations consisted solely of *P*. ×*mohacsyana* and 13.2% solely of *P*. ×*eminens*. Only 1.3% of populations included both hybrids. At last, 9.2% of the populations analysed were mixed (i.e., composed of *P. fruticosa* and one of its hybrids).

## 4 DISCUSSION

Absolute genome size/ploidy level estimation coupled with morphometrics allowed us to identify the *Prunus* species and hybrids concerned (which occurred in 39.5% of populations under study). Homoploid hybridization between the tetraploid parental taxa *Prunus fruticosa* and *P. cerasus* produces tetraploid hybrids (*P*. ×*eminens*). By contrast, heteroploid hybridization between *P. fruticosa* with *P. avium* generates triploids (*P*. ×*mohacsyana*). The frequencies of the two hybrids turned out to be almost equal in the study area. In contrast to previous attempts to assess the rate of hybridization, which were based solely on morphometrics, our multidisciplinary approach revealed a continuous pattern, pointing to introgression.

Flow cytometry has been employed in several descriptive or local studies of *Prunus* (Bennett & Leitch, 1995; Dickson, Arumuganathan, Kresovich, & Doyle, 1992; Macková et al., 2017) and published genome size values fall within the range of measured values presented here. The morphological pattern is also analogous to those found in previous studies (Lepší et al., 2011; Wójcicki, 1991; Wójcicki & Marhold, 1993). Traditionally used morphological characters (abaxial hairs and plant height; Lepší et al., 2011; Wójcicki, 1988) have turned out to be more suitable in the field than the first three characters identified by morphometrics (i.e., distance from the widest part of the lamina to the laminar tip, laminar width and laminar length). However, the morphology‐based determinations of hybrid groups used in previous studies were probably not correct (Chudíková et al., 2012; Lepší et al., 2011; Wójcicki, 1991; Wójcicki & Marhold, 1993). Until now, almost all hybrids had been suggested to be tetraploid (*P*. ×*eminens*; Lepší et al., 2011; Wójcicki, 1991; Wójcicki & Marhold, 1993), but our data show that the frequency of triploid hybrids, which is roughly 50%, had been considerably underestimated. Leaf shape (elliptic Fourier analysis) seems to be a useful complementary trait for distinguishing between pure *Prunus* species and hybrids, and a similar pattern was also detected in one local study of *P. fruticosa* (Czech Republic; Lepší et al., 2011). Thus, based on DNA ploidy level knowledge, the results of previous studies (Chudíková et al., 2012; Lepší et al., 2011; Wójcicki, 1991; Wójcicki & Marhold, 1993) might have to be substantially reevaluated.

### 4.1 Identity of hybrids

Due to the broad range of absolute genome sizes possessed by the parental species and their hybrids, it is almost impossible to distinguish cytometrically between F1 hybrids and their more complex backcrossed counterparts at the homoploid level (i.e., 4× ). Moreover, an intermediate genome size does not necessarily indicate an F1 hybrid. To draw the conclusion that a plant is an F1 hybrid, one has to rule out the possibility that it is a higher or even backcrossed hybrid. Continuous patterns of absolute genome size are nevertheless usually accompanied by enormous morphological variation, and a continuous pattern of data distribution in both absolute genome size and morphology is usually indicative of introgressive hybridization (e.g., Hanušová et al., 2014; Suda et al., 2007; Šmarda & Bureš, 2006). In addition, our correlation analysis and RDA revealed that hybrids with an absolute genome size similar to that of one of their parental taxa are also morphologically close to that parent, which indicates that they are almost certainly backcrossed. A high probability of backcrossing at the tetraploid level is further supported by the substantial fertility of *P. ×eminens* (based on embryology; Macková et al., 2017). By contrast, heteroploid hybridization (i.e., 4× × 2× ) produces comparatively straightforward results due to the existence of an effective triploid block, which constrains backcrossing; this has been proved in the case of triploid *P. ×mohacsyana* (Macková et al., 2017).

### 4.2 Crop‐to‐wild studies and their limitations

Human‐induced hybridization (or even introgression) affects wild plant species in different ways, and there are several cases that are analogous to that of *Prunus fruticosa*. While hybridization of cultivated *Saccharum* L. or *Brassica* L. with wild counterparts does not pose any risk to their wild relatives, hybridization of cultivated *Oryza* L. and *Gossypium* L. has been implicated in the near extinction of certain wild species of rice and cottonseed (Ellstrand et al., 1999).

Studies dealing with crop‐to‐wild gene flow rely on the ability to unequivocally distinguish between wild and cultivated plant forms. In most cases, however, this discrimination is not possible based solely on morphological grounds (e.g., *Malus* Mill. Coart et al., 2006; *Vitis* L. De Andrés et al., 2012). Plant sex might serve as another suitable and conspicuous differential trait (e.g., dioecious wild vs mostly hermaphroditic cultivated forms of *Vitis*; De Andrés et al., 2012). Their discrimination is made markedly easier if a wild species and its cultivated counterpart differ in growth form (e.g., shrub vs tree form in *Prunus*; Delplancke et al., 2012; Macková et al., 2017). The combined approach (absolute genome size/ploidy level and morphology) allowed us to distinguish between wild and cultivated *Prunus* plants. Whereas most studies of crop‐to‐wild introgression deal with rather small datasets (e.g., 237 samples in *Vitis*; De Andrés et al., 2012), our study is based on more than 700 individuals distributed in the Central European region.

Moreover, crop‐to‐wild gene flow studies are frequently complicated by the existence of naturalized individuals (crop progeny), which can be almost indistinguishable from their wild counterparts or introgressants (e.g., *Malus sylvestris* vs *M. domestica*, Coart et al., 2006; *Vitis vinifera* ssp. *sylvestris* (C. C. Gmel.) Hegi vs *V*. *vinifera* ssp. *vinifera* L., De Andrés et al., 2012). In cherries, however, it is quite easy to distinguish the progeny of alien *Prunus cerasus* from indigenous *P. fruticosa* and their hybrids or from introgressants based on their growth form (i.e., their tree vs shrub habitus). Heteroploid hybridization of *P. fruticosa* with *P. avium* is analogous to that in the genus *Malus* because *P. avium* in Europe consists of genuine wild individuals and naturalized individuals, which are almost indistinguishable (Coart et al., 2003; Gross, Henk, Forsline, Richards, & Volk, 2012; Webb, 1968).

### 4.3 Conservation implications

From a species conservation perspective, homoploid hybridization and repeated backcrossing with allochthonous *P. cerasus* accompanied by heteroploid hybridization with autochthonous *P. avium* represent a substantial risk of wild populations of *P. fruticosa*. Plants produced by both types of hybridization may considerably hinder the conservation of wild populations of genuine *P. fruticosa* by competing for resources and suitable niches (analogously as in *Cerastium* L. or *Dianthus* L.; Vít et al., 2014; Vítová et al., 2015) and by decreasing the number of potential mating partners (i.e., demographic swamping; Todesco et al., 2016). The potential for the displacement of *P. fruticosa* is further enhanced by the fact that the two hybrids tend to outgrow it. In contrast to sterile triploid hybrids (Macková et al., 2017), fertile tetraploid hybrids can directly endanger genuine *P. fruticosa* by introgression (i.e., genetic swamping; Todesco et al., 2016). Still, however, some isolated triploid hybrid populations could represent old, partly fertile, spontaneous hybrids with autochthonous *P. avium* (Lepší et al., 2011). Introgression involving triploid hybrids has also been documented in other genera (e.g., *Betula* L. in Iceland; Thórsson, Pálsson, Sigurgeirsson, & Anamthawat‐Jónsson, 2007), so the potential risk that triploid F1 hybrid could participate in further backcrossing cannot be ruled out.

The main practical implication of our results is the necessity to limit the cultivation of both sour and sweet cherries in the vicinity of wild populations of genuine *P. fruticosa* (within a perimeter of at least 1.5 km, as recommended by Boratyński et al., 2003). To this end, it is first necessary to select populations to be protected with high priority (i.e., those which are the most genetically variable—see below).

### 4.4 Genome size analysis as a suitable tool for detecting introgression

The continuous absolute genome size values at the homoploid level, together with the wide morphological variation, suggest repeated backcrossing between parents and hybrids (e.g., Hanušová et al., 2014; Suda et al., 2007; Šmarda & Bureš, 2006). Nevertheless, the obtained pattern, including the impossibility to unequivocally identify F1 hybrids, constitutes only indirect evidence of introgression. However, all other potential explanations (i.e., aneuploidy, differential accumulation of transposable elements, chromosome recombinations, B chromosomes; Bennetzen, Ma, & Devos, 2005; Michael, 2014; Petrov, 2001; Šmarda & Bureš, 2010) are highly unlikely. Our data do not allow us to evaluate population dynamics (changes of hybridization frequency in time) and, particularly, the importance of clonal growth (genetic variation of populations).

The use of molecular markers such as SSRs or RAD‐Seq might provide direct evidence for ongoing introgression and help identify the conservational most valuable (i.e., variable) populations of *P. fruticosa* (Barać et al., 2017; Beghe, Piotti, Satovic, de la Rosa, & Belaj, 2017; McVay, Hipp, & Manos, 2017). However, the complex cytological structure of our data set covering three ploidy levels seriously complicates data analyses. Uncertainty concerning allele dosage in polyploids, an unclear mode of inheritance (Dufresne, Stift, Vergilino, & Mable, 2014) and likely asymmetry in strength of gene flow across ploidies (Kolář, Čertner, Suda, Schönswetter, & Husband, 2017), precludes the use of standard tools for the detection of hybridisation, such as NewHybrids (Anderson & Thompson, 2002).

## 5 CONCLUSIONS

In the wild, genuine *Prunus fruticosa* frequently hybridizes both at the homoploid level (with cultivated *P. cerasus*) and at the heteroploid level (with *P. avium*). Our direct identification and quantification of interspecific hybridization/introgression under natural conditions has confirmed the serious risk of ongoing demographic and genetic swamping, as 39.5% of the populations we studied are of hybrid origin. Moreover, homoploid introgressive hybridization poses a substantial conservation threat because *P. cerasus* is alien to the European flora. Maintenance of a diverse and heterogeneous *P. fruticosa* gene pool is essential for *Prunus* breeding programmes as well as for the species’ protection. A future conservation genetic investigation should focus on the identification of the most valuable (i.e., the most genetically variable) populations of genuine *P. fruticosa*.

## CONFLICT OF INTEREST

None declared

## DATA ARCHIVING STATEMENT

Data available from the Dryad Digital Repository: https://doi.org/10.5061/dryad.v7k54df
