# Supplementary figures and images for: Crop‐to‐wild hybridization in cherries—Empirical evidence from Prunus fruticosa
Source: Evol Appl. 2018 Jul 26;11(9):1748–59. doi: 10.1111/eva.12677 (PMC6183504; doi:10.1111/eva.12677)

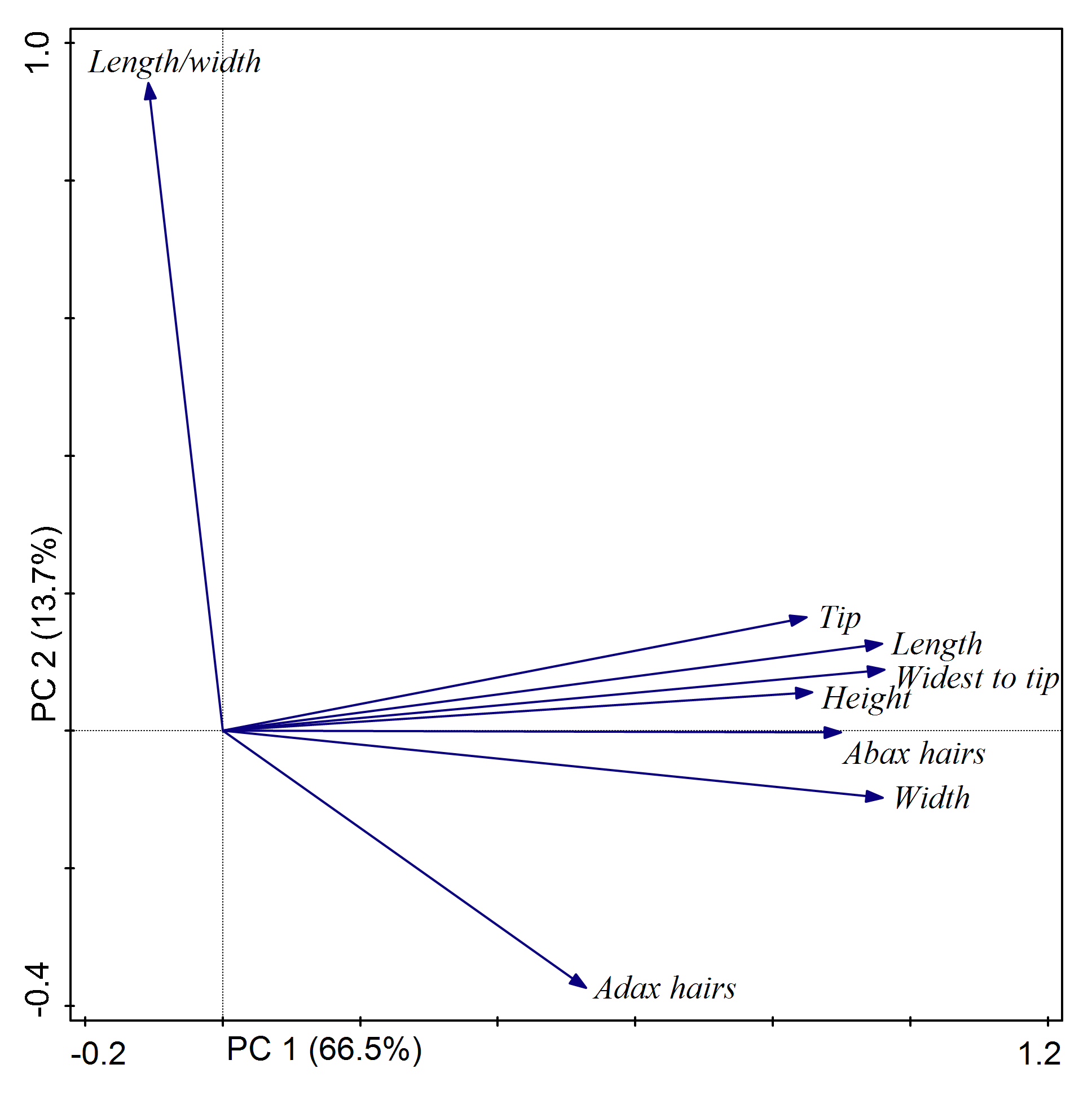

Supplement: Supplementary file 1 [file EVA-11-1748-s001.tif]

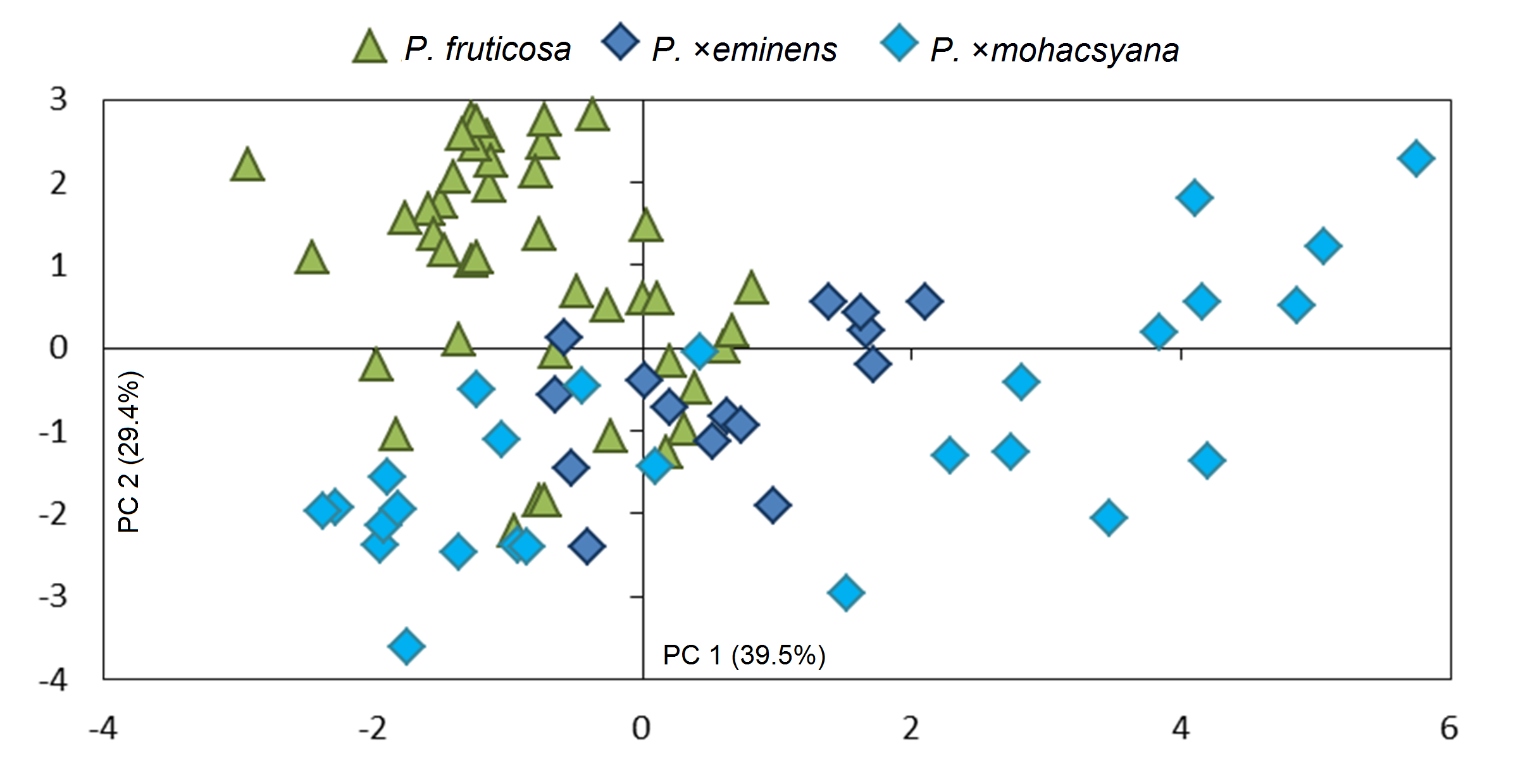

Supplement: Supplementary file 2 [file EVA-11-1748-s002.tif]

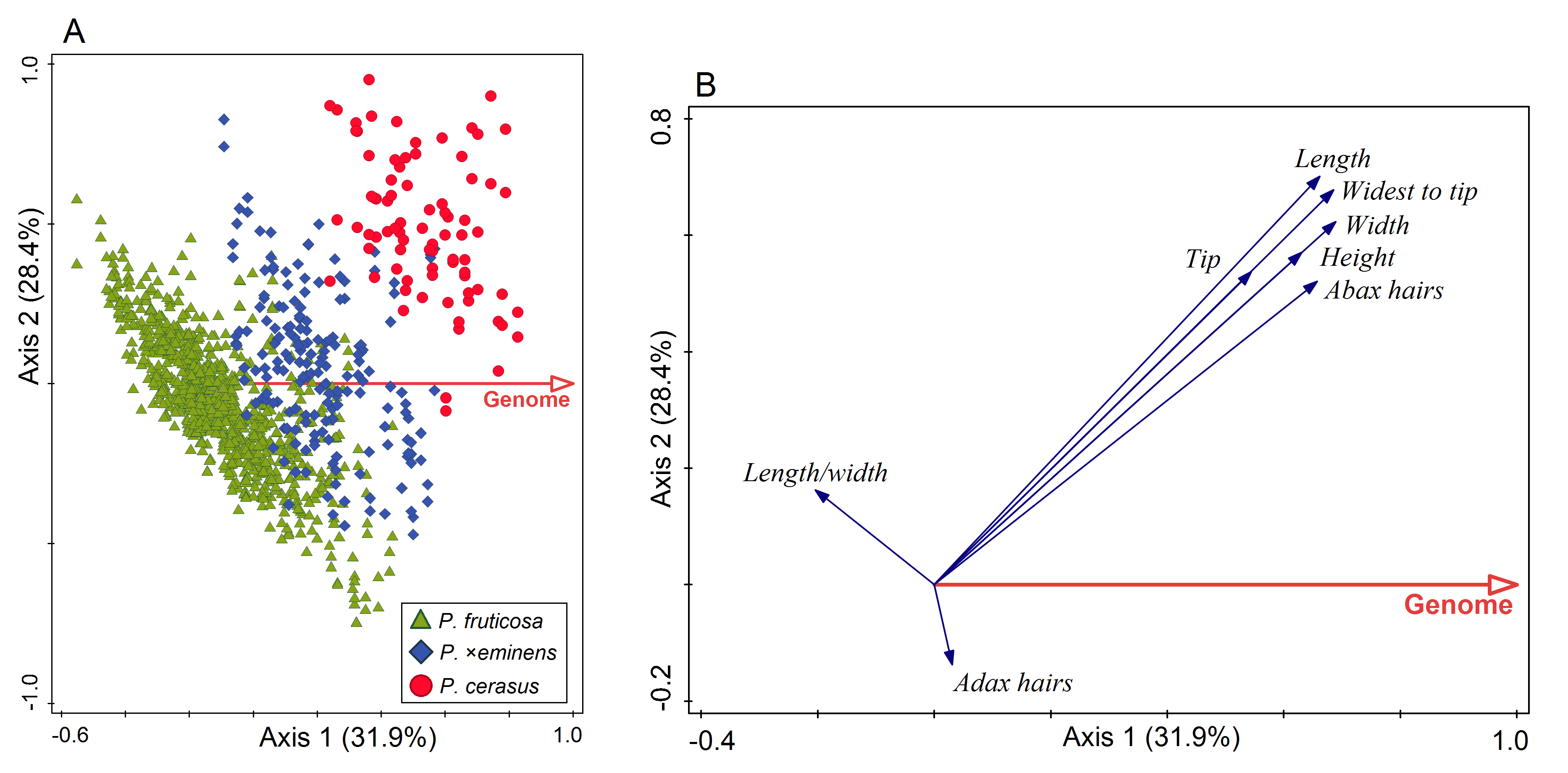

Supplement: Supplementary file 3 [file EVA-11-1748-s003.tif]
